# Sleep deprivation aggravates brain injury after experimental subarachnoid hemorrhage via TLR4-MyD88 pathway

**DOI:** 10.18632/aging.202503

**Published:** 2021-01-21

**Authors:** Ye-Ping Xu, Yun-Na Tao, You-Ping Wu, Jing Zhang, Wei Jiao, Yu-Hai Wang, Tao Chen

**Affiliations:** 1 Department of Neurosurgery, The 904 Hospital of PLA Joint Logistic Support Force, Medical School of Anhui Medical University, Wuxi 214044, Jiangsu, China; 2 Department of Nursing, The 904 Hospital of PLA Joint Logistic Support Force, Medical School of Anhui Medical University, Wuxi 214044, Jiangsu, China

**Keywords:** sleep deprivation, toll-like receptor 4, subarachnoid hemorrhage, myeloid differentiation primary response protein 88

## Abstract

Subarachnoid hemorrhage (SAH) is a life-threatening cerebrovascular disease, and most of the SAH patients experience sleep deprivation during their hospital stay. It is well-known that sleep deprivation is one of the key components of developing several neurological disorders, but its effect on brain damage after SAH has not been determined. Therefore, this study was designed to evaluate the effect of sleep deprivation using an experimental SAH model in rats. Induction of sleep deprivation for 24 h aggravated the SAH-induced brain damage, as evidenced by brain edema, neuronal apoptosis and activation of caspase-3. Sleep deprivation also worsened the neurological impairment and cognitive deficits after SAH. The results of immunostaining and western blot showed that sleep deprivation increased the activation of microglial cells. In addition, sleep deprivation differently regulated the expression of anti-inflammatory and pro-inflammatory cytokines. The results of immunofluorescence staining and western blot showed that sleep deprivation markedly increased the activation of Toll-like receptor 4 (TLR4) and myeloid differentiation primary response protein 88 (MyD88). Mechanically, treatment with the TLR4 inhibitor TAK-242 or the MyD88 inhibitor ST2825 significantly attenuated the brain damage and neuroinflammation induced by sleep deprivation after SAH. In conclusion, our results indicate that sleep deprivation aggravates brain damage and neurological dysfunction following experimental SAH in rats. These effects were mediated by the activation of the TLR4-MyD88 cascades and regulation of neuroinflammation.

## INTRODUCTION

Spontaneous subarachnoid hemorrhage (SAH), commonly caused by aneurysm rupture, is a life-threatening cerebrovascular disorder of the central nervous system. Although a decreased mortality rate achieved recently due to great progress in microsurgery and endovascular embolization, various neurosurgical and medical complications, such as delayed brain ischemia cerebrospinal fluid leak, pneumonia or deep vein thrombosis, occur in more than half of the patients [1]. Both clinical and experimental results indicate that neuroinflammation is one of the critical factors involved in both early neuronal injury and delayed vasospasm following SAH [2]. Thus, inhibiting inflammatory responses via gene intervention technology or pharmacological inhibitors has been demonstrated to be an ideal therapeutic strategy against brain injury induced by SAH [3, 4].

Sleep is a natural state of the human body that enables us to perform everyday activities properly. Sleep deprivation, defined as complete loss of sleep throughout a time period, is very common in specific occupations, such as doctors, nurses, police, soldiers, firefighters and other similar round the clock workers [5, 6]. Although sporadic exposure to sleep deprivation will not cause long-term deficits on brain function, prolonged sleep deprivation will result in acute and chronic damage to cerebral function [7]. Rapid eye movement (REM) sleep deprivation was found to increase brain excitability through the noradrenaline mediated stimulation of Na^+^-K^+^ ATPase activity [8]. Under pathological conditions, sleep deprivation could destroy the repair system, block metabolite clearance mechanism in the brain, inducing oxidative stress and neuronal injury [9]. A previous study showed that sleep deprivation exacerbated the concussive head injury induced brain pathology, and sleep deprivation has been demonstrated to be an important risk factor for stroke [10, 11]. However, the effect of sleep deprivation on SAH has not been deeply investigated. Here, we investigated the role of sleep deprivation in brain damage following experimental SAH in rats.

## RESULTS

### Sleep deprivation aggravates brain damage after SAH

**Figure 1:**
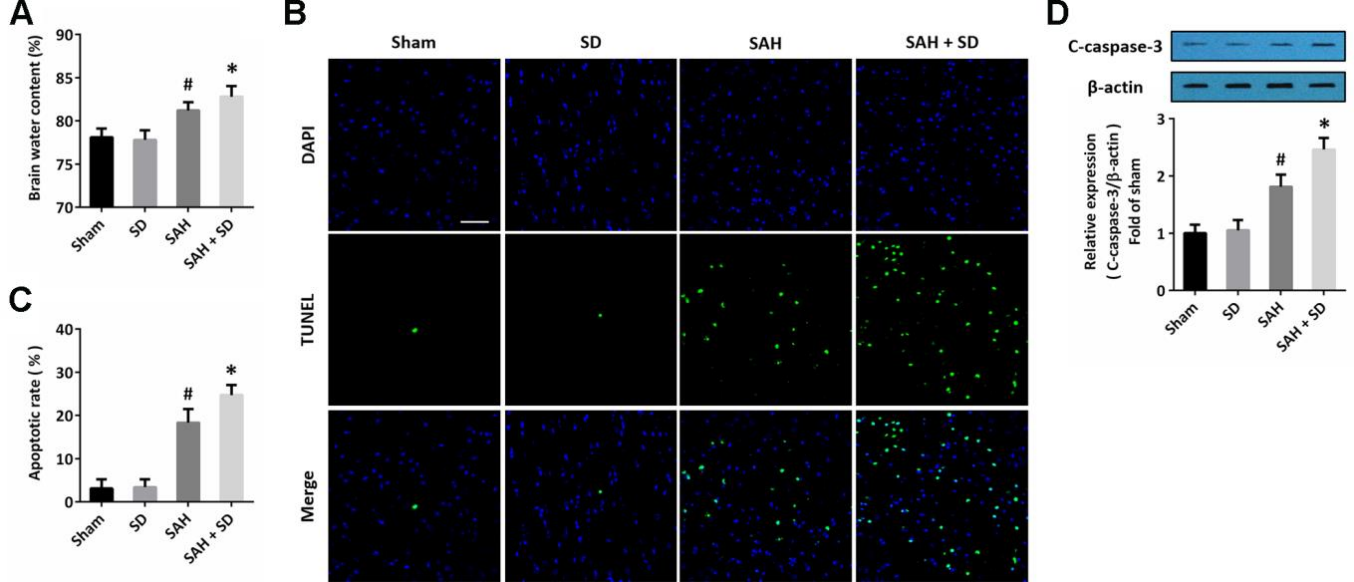
**Sleep deprivation aggravates brain damage after SAH.** (**A**) Brain water content assay shows that sleep deprivation aggravated brain edema after SAH. (**B**, **C**) TUNEL staining (**B**) and quantification (**C**) show that sleep deprivation increased apoptosis after SAH. Scale bar, 50 μm. (**D**) Western blot shows that sleep deprivation increased expression of cleaved-caspase-3 (C-caspase-3) after SAH. The data was represented as means ± SEM. ^#^*p* < 0.05 vs. Sham group and ^*^p < 0.05 vs. SAH group.

To investigate the effect of sleep deprivation on brain damage after SAH, animals were treated with SAH and subjected to sleep deprivation for 24 h. The brain edema was measured by the wet-dry method, and the results showed that SAH caused an in increase in brain water content, which was aggravated by sleep deprivation (Figure 1A). Next, we detected the apoptosis in the brain section using TUNEL staining (Figure 1B), and the SAH-induced increase in apoptotic rate was markedly increased by sleep deprivation (Figure 1C). As shown in Figure 1D, the caspase-3 activation after SAH was further promoted by sleep deprivation.

### Sleep deprivation worsens SAH-induced neurological dysfunction

**Figure 2:**
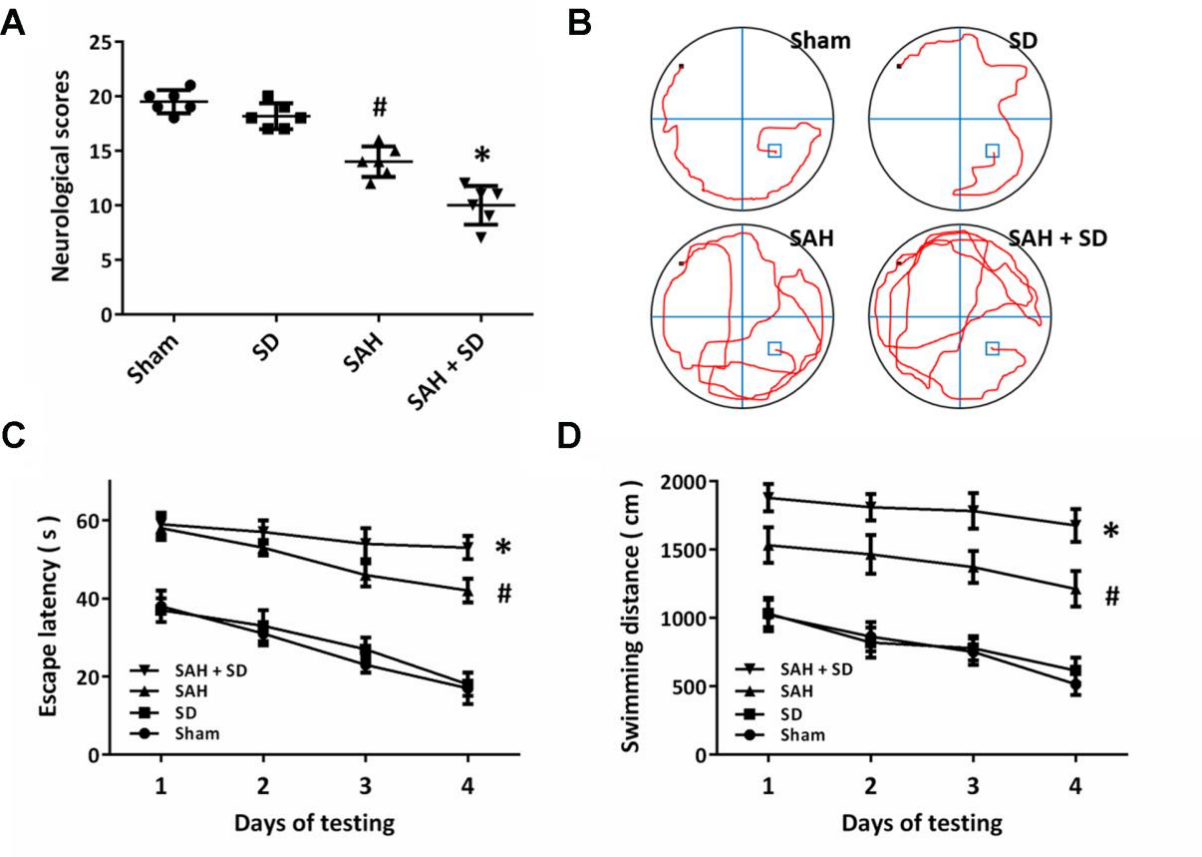
**Sleep deprivation worsens SAH-induced neurological dysfunction.** (**A**) Neurological score assay shows that sleep deprivation aggravated neurological impairment induced by SAH. (**B**–**D**) MWM assay (**B**) and quantification (**C**, **D**) show that sleep deprivation increased escape latencies (**C**) and swimming distance (**D**) over 4 days. The data was represented as means ± SEM. ^#^*p* < 0.05 vs. Sham group and ^*^p < 0.05 vs. SAH group.

We used the composite Garcia neuro-score to determine the neurological impairment after SAH (Figure 2A). The obvious neurological dysfunction was observed in SAH-treated animals, whereas the neurological score in SAH + SD group was lower than that in SAH group. As shown in Figure 2B, the cognitive function was determined by the Morris water maze. Sleep deprivation aggravated the impairments in cognitive behavior as evidenced by the decreased escape latency (Figure 2C). Rats in SAH + SD group showed markedly longer swimming distance than that in SAH group (Figure 2D).

### Sleep deprivation regulates neuroinflammation after SAH

**Figure 3:**
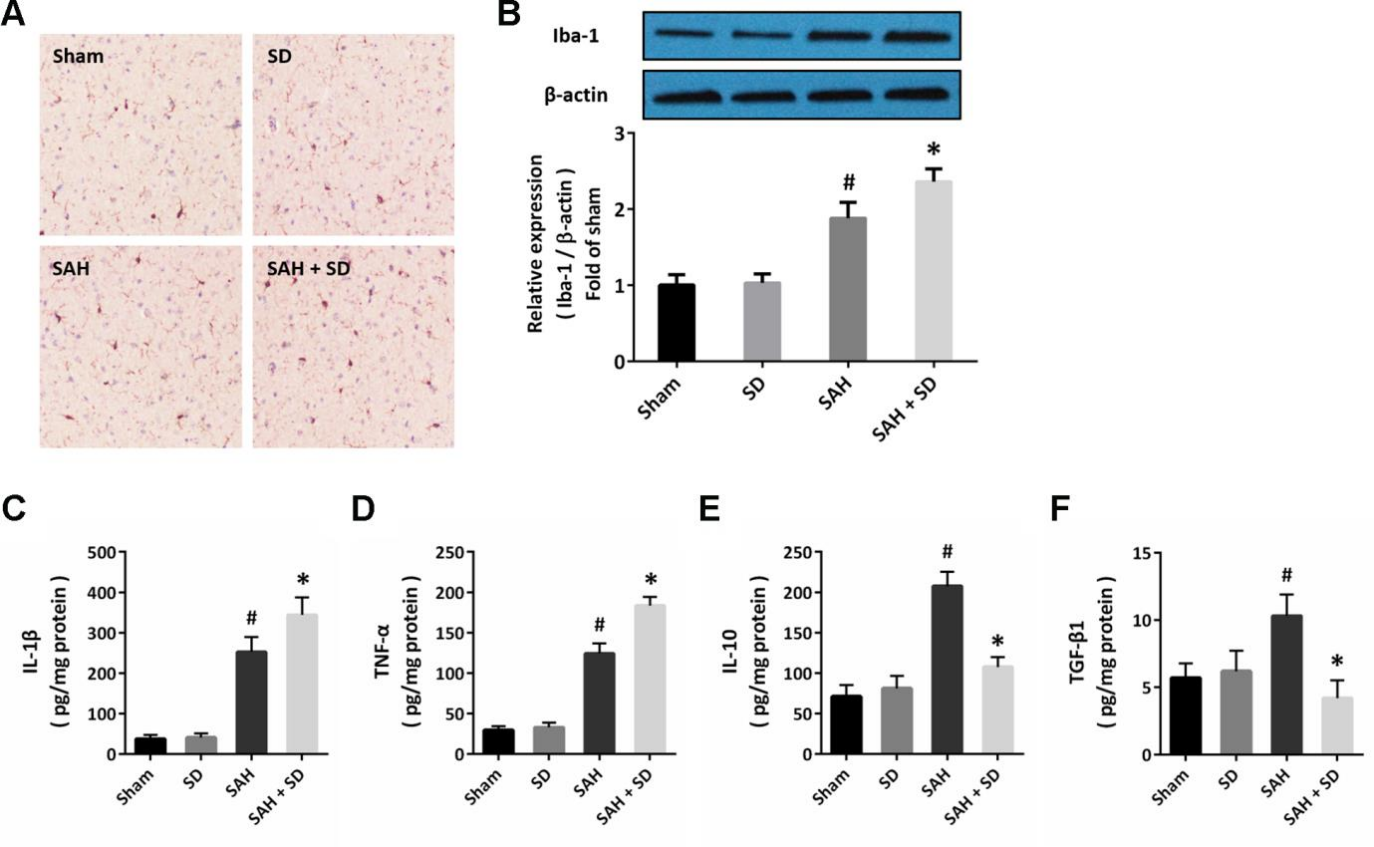
**Sleep deprivation regulates neuroinflammation after SAH.** (**A**) Cytochemistry assay shows that sleep deprivation increased the number of Iba-1 positive cells after SAH. (**B**) Western blot shows that sleep deprivation increased the expression of Iba-1 after SAH. (**C**–**F**) ELISA shows that sleep deprivation increased the levels of IL-1β (**C**) and TNF-α (**D**), but decreased the levels of IL-10 (**E**) and TGF-β1 (**F**) after SAH in rats. The data was represented as means ± SEM. ^#^*p* < 0.05 vs. Sham group and ^*^p < 0.05 vs. SAH group.

Next, we performed immunostaining using the Iba-1 antibody to detect the activation of microglial cells in brain section (Figure 3A). The number of Iba-1 positive cells was much higher in SAH + SD group than that in SAH group. In congruent, sleep deprivation significantly increased the expression of Iba-1 after SAH (Figure 2B). In addition, the ELISA assay was also performed to determine the expression of inflammatory cytokines. The results showed that sleep deprivation further increased the expression of IL-1β (Figure 3C) and TNF-α (Figure 3D), but decreased the expression of IL-10 (Figure 2E) and TGF-β1 (Figure 2F) after SAH.

### Sleep deprivation activates TLR4 signaling after SAH

**Figure 4:**
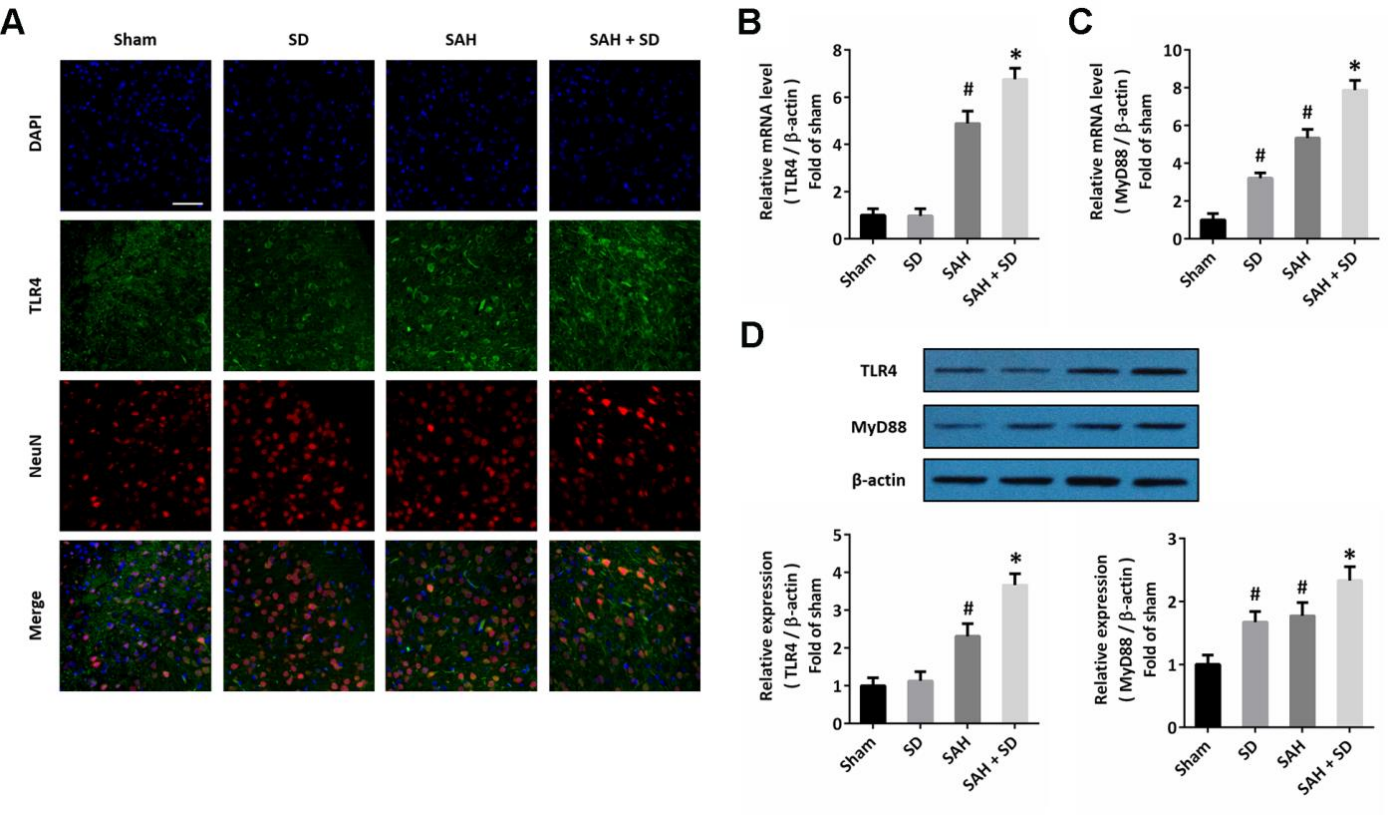
**Sleep deprivation activates TLR4 signaling after SAH.** (**A**) Immunofluorescence staining shows that sleep deprivation increased TLR4 expression in neurons after SAH. Scale bar, 50 μm. (**B**, **C**) RT-PCR show that sleep deprivation increased the mRNA levels of TLR4 (**B**) and MyD88 (**C**) after SAH. (**D**) Western blot shows that sleep deprivation increased the expression of TLR4 and MyD88 after SAH. The data was represented as means ± SEM. ^#^*p* < 0.05 vs. Sham group, ^*^p < 0.05 vs. SAH group.

To investigate the possible molecular mechanism underlying our data, we performed immunofluorescence staining using the TLR4 antibody, and we found that sleep deprivation increased the expression of TLR4 after SAH, especially in neurons (Figure 4A). The results of RT-PCR showed that sleep deprivation significantly increased the mRNA levels of both TLR4 (Figure 4B) and MyD88 (Figure 4C) after SAH. As shown in Figure 4D, the increased expression of TLR4 and MyD88 protein induced by SAH was further enhanced by sleep deprivation.

### Involvement of TLR4-MyD88 pathway in sleep deprivation-induced aggravation of brain injury after SAH

**Figure 5:**
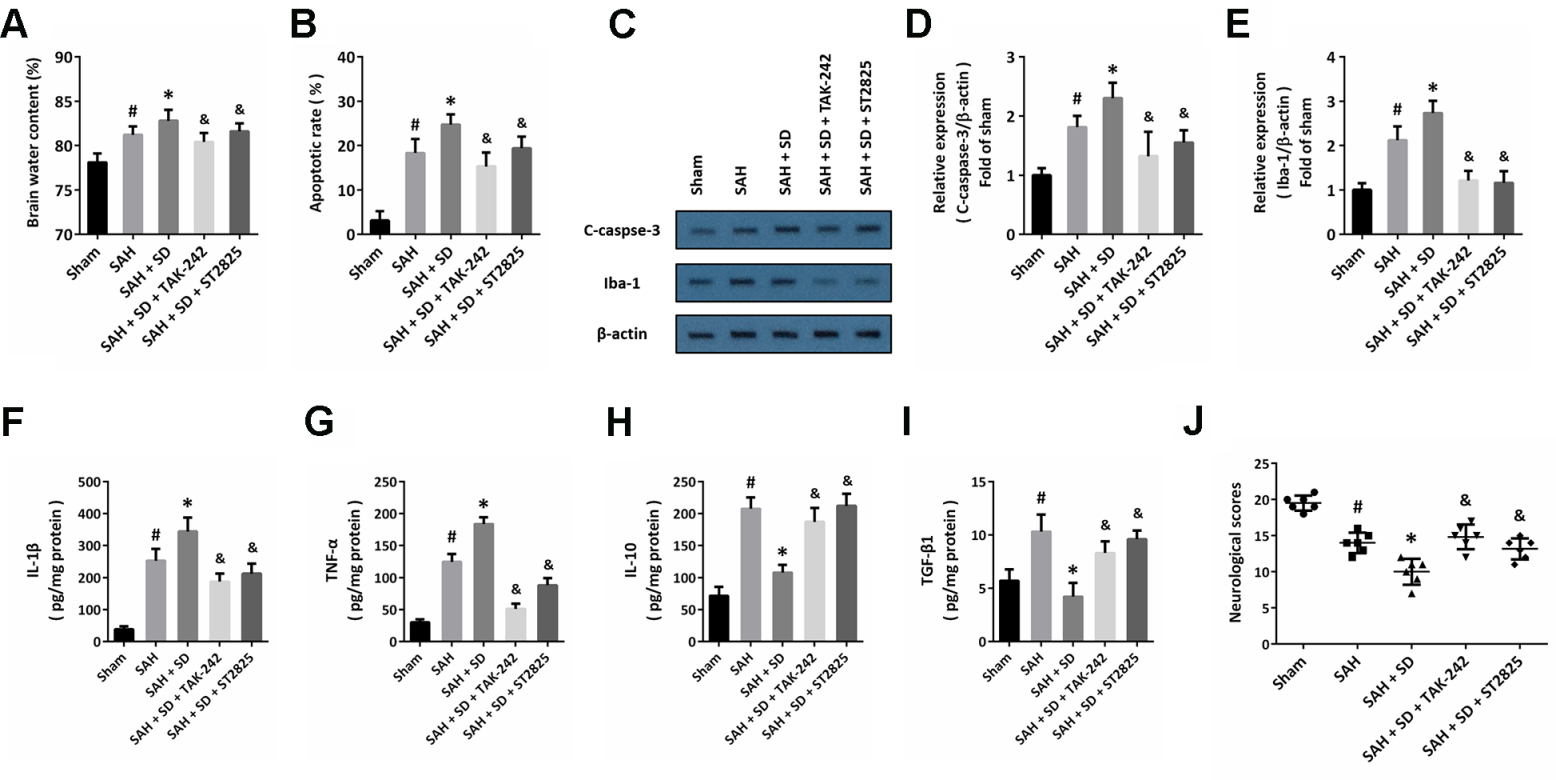
**Involvement of TLR4-MyD88 pathway in sleep deprivation-induced aggravation of brain injury after SAH.** (**A**) Brain water content assay shows that aggravation of brain edema induced by sleep deprivation after SAH was prevented by TAK-242 and ST2825. (**B**) TUNEL staining shows that the increase in apoptosis induced by sleep deprivation after SAH was inhibited by TAK-242 and ST2825. (**C**–**E**) Western blot (**C**) and quantification (**D**, **E**) show that the increased expression of C-caspase-3 (**D**) and Iba-1 (**E**) induced by sleep deprivation after SAH was prevented by TAK-242 and ST2825. (**F**–**I**) ELISA shows that the increased levels of IL-1β (**F**) and TNF-α (**G**) and decreased the levels of IL-10 (**H**) and TGF-β1 (**I**) induced by sleep deprivation after SAH were reversed by TAK-242 and ST2825. (**J**) Neurological score assay shows that aggravation of neurological impairment induced by sleep deprivation after SAH was prevented by TAK-242 and ST2825. The data was represented as means ± SEM. ^#^*p* < 0.05 vs. Sham group, ^*^p < 0.05 vs. SAH group and ^&^*p* < 0.05 vs. SAH+SD group.

To further confirm the involvement of the TLR4-MyD88 pathway, animals were treated with TAK-242 or ST2825 to block the activation of TLR4 or MyD88, respectively. The data showed that the sleep deprivation-induced aggravation of brain injury, as evidenced by increased brain water content (Figure 5A), apoptosis (Figure 5B) and activation of caspase-3 (Figure 5C, 5D), were significantly attenuated by TAK-242 or ST2825. In addition, the increased activation of Iba-1 (Figure 5C–5E), increased levels of IL-1β (Figure 5F) and TNF-α (Figure 5G), as well as the decreased levels of IL-10 (Figure 5H) and TGF-β1 (Figure 5I) induced by SAH and sleep deprivation were all markedly reversed by TAK-242 or ST2825. As shown in Figure 5J, a similar result on neurological score was also observed.

## DISCUSSION

Sleep deprivation has profound effects on brain function from the cellular level to the integrated functions, including behavior, memory and cognitive performance [12]. In the present study, our results indicate that sleep deprivation aggravates brain damage following experimental SAH via promoting neuroinflammatory responses. We found that (a) sleep deprivation increases brain edema and neuronal apoptosis induced by SAH; (b) sleep deprivation worsens SAH-induced neurological dysfunction and MWM performance; (c) sleep deprivation increases the Iba-1 activation and regulates the expression of inflammatory cytokines; (d) sleep deprivation increases the expression of TLR4 and MyD88 following SAH; and (e) the inhibitors of TLR4 signaling pathway prevents the aggravation of sleep deprivation on brain injury and neuroinflammation after experimental SAH in rats.

Most patients with SAH require monitoring and treatment in the intensive care unit (ICU), where approximately 80% of the patients experience sleep deprivation during their hospital stay [13]. Accumulating evidence clearly shows that sleep deprivation is one of the key components of developing several neurological diseases, such as stroke, Alzheimer’s disease (AD), Parkinson’s disease (PD), epilepsy and brain trauma. A previous study showed that sleep deprivation exacerbated the concussive head injury induced brain pathology via promoting blood-brain barrier breakdown [10]. However, Martinez-Vargas et al. showed that 24 h of total sleep deprivation after TBI alleviated brain damage and enhances the recovery of the neurological function in rats [14]. The authors speculated that these protective effects might be related to a circadian rhythm disturbance, and we thought that this contradiction could be explained by the different methods to induce sleep deprivation and the duration of the sleep deprivation period. In the present study, total sleep deprivation for 24 h after SAH was found to aggravate brain damage, which was clearly demonstrated by the increased brain edema, neuronal apoptosis and activation of caspase-3. Previous studies have shown that sleep deprivation could elevate brain and body temperature, which worsen the clinical conditions by changing the neuronal membrane properties and spike activity [15]. In addition, sleep deprivation was found to exert detrimental effects via altering glucose metabolism, decreasing insulin sensitivity and increasing hypertension [16]. Thus, sleep deprivation might be one of the mechanisms underlying secondary brain injury following SAH that affects the outcome of the patients.

Inflammation is the fundamental response of tissues to injury and is generally required for the healing process. After SAH, subarachnoid blood and the degraded products, such as hemoglobin, methemoglobin, oxyhemoglobin heme and hemin, can in turn trigger the inflammation cascades, which contribute to the angiographic cerebral vasospasm, delayed cerebral ischemia, and systemic complications [3]. In addition, the systemic inflammatory syndrome usually occurs after SAH, with non-infectious fever and systematic complications. Previous studies have shown that sleep disturbance is closely related to the risk and all-cause mortality of inflammatory diseases [17]. The increased expression of C-reactive protein (CRP) and interleukin-6 (IL-6), two important circulating markers of inflammation, were found after sleep deprivation, and has been shown to predict cardiovascular events, hypertension and type two diabetes [18–20]. Thus, inflammatory response is the common mechanism induced by SAH and sleep deprivation, indicating that anti-inflammatory strategy might be an ideal candidate for the treatment of these patients. Our present data showed that SAH enhanced the activation of microglial cells, which was further promoted by sleep deprivation in rats. The SAH-induced neuroinflammation was demonstrated to be characterized by activation of microglial cells, which in turn lead to the upregulation of cellular adhesion molecules, recruitment of immune cells and release of inflammatory cytokines [2]. Previous studies have shown that sleep deprivation results in endothelial production of inflammatory cytokines, partially due to the increase in endothelial shear stresses induced by the elevated blood pressure [21, 22]. It has been demonstrated that both exogenous administration and gene transfer of IL-10 and TGF-β1 protected against brain injury [23–26]. We found that sleep deprivation aggravated the brain inflammatory response after SAH via differently regulating the levels of pro-inflammatory and anti-inflammatory cytokines. These pro-inflammatory effects were accompanied by the increased neurological dysfunction. In congruent, a previous study showed that sleep deprivation increased the microglial cells activation and promoted the production of pro-inflammatory cytokines levels in rats [27].

Toll-like receptors (TLRs) are a family of receptors involved in pathogen recognition and host defense, which can be activated by the endogenous (damage-associated molecular patterns, DAMPs) or the exogenous (pathogen-associated molecular patterns, PAMPs) ligands [28]. TLRs are widely expressed in various mammalian immune and non-immune cells, mostly in microglial cells, but also in astrocytes and neurons [29–31]. Activation of TLR4 has been shown to trigger and modulate neuroinflammatory responses and contribute to secondary brain injury after both acute and chronic neurological diseases [32]. Previous studies using the mouse SAH model showed that activation of TLR4 promoted neuronal apoptosis via microglial- and MyD88-dependent mechanism in the early phase of SAH, whereas the toll receptor-associated activator of interferon (TRIF)-dependent but microglia-independent mechanism was involved in the late phase [33]. In the present study, the increased expression of TLR4 and MyD88 was detected by western blot after SAH, which was further enhanced by sleep deprivation. In addition, the brain edema, neuronal apoptosis and neurological dysfunction induced by SAH and/or sleep deprivation were markedly attenuated by treatment with TAK-242 or ST2825. TAK-242 is a specific TLR4 inhibitor with low molecular weight, which binds to Cys747 in the intracellular domain of TLR4 [34]. The potent and selective MyD88 inhibitor ST2825 interferes with recruitment of IL-1R associated kinase (IRAK)-1 and IRAK-4 by MyD88, leading to the inhibition of downstream mitogen-activated protein kinases (MAPKs) and NF-κB signaling pathways [35]. Thus, our present data suggest that the aggravation of brain damage induced by sleep deprivation after SAH was at least partially mediated by the TLR4-MyD88 pathway. Intriguingly, the results of immunostaining showed that the increased expression of TLR4 was mainly located at neurons as evidenced by the staining with NeuN antibody. The functional implication of TLRs expression in neurons has not been well investigated, but it was thought that TLR4 activation might contribute to neuronal plasticity and development in neurons [36]. The exact biological function of TLR4 in different cell types in the brain needs to be further determined.

There are some limitations to this study. First, various factors affect the sleep quality of patients with SAH, such as medications, preexisting sleep conditions, critical complications after surgery and cerebral perfusion, and many approaches may be done to promoting sleep quality, including nursing care plans, medication regimens, as well as sleep hygiene [37, 38]. These complicated conditions cannot be fully mimicked by the experimental sleep deprivation model used in the present study. We will do more experiments using different sleep deprivation animal models and using different sleep deprivation duration and treatment intervals in the future. In addition, the activation of TLR4 signaling cascades can be observed in both neurons and glial cells, and the interactions between neurons and glial cells are key factors that determine the neurological function under neuroinflammation [39, 40]. In our experimental SAH model, the expression of TLR4 and MyD88 was detected in the whole brain tissue homogenates using western blot. Some more experiments using *in vitro* models, such as primary cultured microglial cells or neuron-glial co-cultures, will be helpful for confirming the mechanisms observed here.

In summary, our results indicate that sleep deprivation aggravates brain damage and neurological dysfunction following experimental SAH in rats. These effects were dependent on the activation of the TLR4-MyD88 signaling cascades and regulation of neuroinflammation.

## MATERIALS AND METHODS

### Subjects

All experimental procedures were approved by the Animal Care and Use Committee of Anhui Medical University. Male Sprague-Dawley (SD) rats, aged at 6-8 weeks and weighing 300-330 g, were obtained from Animal Center of Anhui Medical University (Hefei, China).

### SAH models

To mimic SAH-associated brain injury *in vivo*, the single-hemorrhage model induced by injecting autologous blood into the prechiasmatic cistern was established. Adult SD rats were anesthetized by 10% chloral hydrate in the stereotaxic frame, and 0.35 ml fresh arterial blood was slowly injected into the prechiasmatic cistern within 20 s. Bone wax was used to plug the burr hole in the skull before inserting the needle to prevent the leakage of the cerebrospinal fluid during modeling. The animal’s core body temperature was maintained at 37.5 ± 0.5° C using a heating pad.

### Sleep deprivation

A tank with a diameter and height of 50 cm and 50 cm was used to induce sleep deprivation as previously described with some modification [41].

### Experimental design

Experimental 1: seventy-two SD rats were randomly included into four groups of eighteen in each group, as follow: Sham group, sleep deprivation group (SD), SAH group and SAH + SD group. In every group, six animals were used for measuring brain edema; six animals were used for western blot, immunostaining and ELISA; and six animals were used for neurological scoring and Morris water maze (MWM).

Experimental 2: ninety SD rats were randomly included into five groups of eighteen in each group, as follow: Sham group, SAH group, SAH + SD group, SAH + SD + TAK-242 group (intraperitoneally treated with the TLR4 inhibitor TAK-242 at 3 mg/kg) and SAH + SD + ST2825 group (intracerebroventricularly treated with the MyD88 inhibitor ST2825 at 10 μg). In each group, six rats were used for measuring brain edema; six animals were used for TUNEL staining; and six animals were used for neurological scoring.

### Brain edema measurement

The wet-dry method was used to determine SAH-induced brain edema by measuring brain water content following a standard protocol.

### TUNEL staining

Apoptotic cell death within brain sections was determined by measuring DNA strand breaks in the nuclei using TUNEL staining. Coronal sections obtained from rats suffered from SAH and/or sleep deprivation were permeabilized by proteinase K solution and incubated with fluorescein TUNEL reagent mixture following the manufacturer’s protocol (Promega, USA). The apoptotic rate was calculated after counting the number of TUNEL-positive cells and the DAPI-positive cells.

### Neurological scoring

At 24 h after sleep deprivation, neurological function of the rats was determined using the composite Garcia neuro-score [42].

### MWM assay

The MWM test was performed to determine the spatial learning and memory function as previously described [43].

### ELISA assay

To investigate the levels of inflammatory cytokines, rats were sacrificed at 24 h after SD and the brain tissue homogenates were obtained. The concentrations of IL-1β, TNF-α, IL-10 and TGF-β1 were measured using specific ELISA kits according to the manufacturers' instructions (Boster Biological Technology, Wuhan, China).

### Immunofluorescence staining

The coronal brain sections were permeabilized with 3% Triton X-100 for 10 min, blocked with 10% normal donkey serum in phosphate buffer saline (PBS) for 60 min and incubated overnight with the following primary antibodies at 4° C: TLR4 (sc-293072, Santa Cruz, 1:200) and NeuN (#24307, Cell Signaling, 1:500). Then, brain sections were incubated with fluorescent donkey anti-rabbit secondary antibodies, including Alexa Fluor 488 for TLR4 (green) and Alexa Fluor 594 for NeuN (red) obtained from Molecular Probes (1:500) at 37° C for 2 h.

### Real-time RT-PCR

Total RNA was prepared from brain tissue homogenates with the Trizol Reagent method [44]. The mRNA levels were determined by real-time RT-PCR, and the primer set is: *TLR4*: forward, 5'-ATG GCA TGG CTT ACA CCA CC-3'; reverse, 5'-GAG GCC AAT TTT GTC TCC ACA-3'; *MyD88*: forward, 5'-TTC TCC AAC GCT GTC CTG TC-3'; reverse, 5'-AAC TGA GAT GTG TGC CCA GG-3'; *β-actin*: forward, 5’-AGG GAA ATC GTG CGT GAC-3’; reverse, 5’-CGC TCA TTG CCG ATA GTG-3’. The relative expression value was normalized to the expression value of β-actin.

### Western blot analysis

After various treatments, cortical homogenates were obtained for western blot analysis. Commercial primary antibodies were used: Iba-1 (#17198, Cell Signaling, 1:1000), TLR4 (sc-293072, Santa Cruz, 1:200), cleaved-caspase-3 (#9664, Cell Signaling, 1:600), MyD88 (#4283, Cell Signaling, 1:800) and β-actin (ab8226, Abcam, 1:2000).

### Statistical analysis

The data was represented as means ± SEM. All statistical analyzes were performed using the SPSS 16.0. The one-way analysis of variance (ANOVA) followed by Bonferroni’s multiple comparisons (multiple groups) or unpaired *t* test (two groups) were used. A value of *p* < 0.05 was considered statistically significant.
